# Overexpression of Cancer-Associated Stem Cell Gene *OLFM4* in the Colonic Epithelium of Patients With Primary Sclerosing Cholangitis

**DOI:** 10.1093/ibd/izab025

**Published:** 2021-02-11

**Authors:** Mastura Neyazi, Sraddha S Bharadwaj, Samuel Bullers, Zofia Varenyiova, Oxford IBD Cohort Study Investigators, Simon Travis, Carolina V Arancibia-Cárcamo, Fiona Powrie, Alessandra Geremia

**Affiliations:** 1 Translational Gastroenterology Unit, Nuffield Department of Clinical Medicine, Experimental Medicine Division, and National Institute for Health Research Oxford Biomedical Research Centre, Oxford University Hospitals National Health Services Foundation Trust, John Radcliffe Hospital, University of Oxford, Oxford, UK; 2 Kennedy Institute of Rheumatology, Nuffield Department of Orthopaedics, Rheumatology and Musculoskeletal Science, University of Oxford, Oxford, UK

**Keywords:** primary sclerosing cholangitis, inflammatory bowel disease, organoids, epithelium

## Abstract

**Background:**

To examine immune-epithelial interactions and their impact on epithelial transformation in primary sclerosing cholangitis–associated ulcerative colitis (PSC-UC) using patient-derived colonic epithelial organoid cultures (EpOCs).

**Methods:**

The EpOCs were originated from colonic biopsies from patients with PSC-UC (n = 12), patients with UC (n = 14), and control patients (n = 10) and stimulated with cytokines previously associated with intestinal inflammation (interferon (IFN) γ and interleukin (IL)-22). Markers of cytokine downstream pathways, stemness, and pluripotency were analyzed by real-time quantitative polymerase chain reaction and immunofluorescence. The *OLFM4* expression in situ was assessed by RNAscope and immunohistochemistry.

**Results:**

A distinct expression of stem cell–associated genes was observed in EpOCs derived from patients with PSC-UC, with lower expression of the classical stem-cell marker *LGR5* and overexpression of *OLFM4,* previously associated with pluripotency and early stages of neoplastic transformation in the gastrointestinal and biliary tracts. High levels of *OLFM4* were also found ex vivo in colonic biopsies from patients with PSC-UC. In addition, IFNγ stimulation resulted in the downregulation of *LGR5* in EpOCs, whereas higher expression of *OLFM4* was observed after IL-22 stimulation. Interestingly, expression of the IL-22 receptor, *IL22RA1,* was induced by IFNγ, suggesting that a complex interplay between these cytokines may contribute to carcinogenesis in PSC-UC.

**Conclusions:**

Higher expression of *OLFM4,* a cancer stemness gene induced by IL-22, is present in PSC-UC, suggesting that IL-22 responses may result in alterations of the intestinal stem-cell niche in these patients.

## INTRODUCTION

Primary sclerosing cholangitis (PSC) is a chronic progressive disorder of the hepatobiliary system characterized by inflammation, fibrosis, and stricturing of the intrahepatic and extrahepatic bile ducts, leading to liver cirrhosis. There is no cure for PSC, and prognosis remains poor. It is associated with inflammatory bowel disease (IBD) in 60% to 80% of patients, of whom 80% are diagnosed with ulcerative colitis (UC).^1^ However, it is now recognized that colitis in PSC-IBD has distinct features, including more frequent pancolonic, right-sided patchy inflammation, rectal sparing and “backwash” ileitis, and an usually quiescent course.^2^ Most important, patients with primary sclerosing cholangitis–associated ulcerative colitis (PSC-UC) have a 4-fold increased risk of developing colorectal cancer (CRC) compared with patients with UC alone, and 10 times more than the general population.^3^ These cancers more frequently arise from the right colon in PSC, suggesting distinct pathogenetic mechanisms and a possible link with the underlying inflammatory process. Patients with PSC are also at increased risk of hepatobiliary malignancies, particularly cholangiocarcinoma (cumulative lifetime incidence, 10% to 30%), gall bladder adenoma, dysplasia, and carcinoma. Patient prognosis remains poor, with mortality rates as high as 30% at 6 years from diagnosis, with cancer as the main cause (40% to 50% of deaths), followed by liver failure (30% to 40%).^4^ Unfortunately, no medical therapy has proven to be effective, and liver transplant remains the only treatment available.

The pathogenesis of PSC remains unknown. Several hypotheses have been put forward, such as an aberrant homing of gut-derived memory T cells to the liver and bacterial translocation secondary to increased intestinal permeability (the “leaky gut” hypothesis) contributing to the pathogenesis of the disease.^5, 6^ Genetic studies have identified multiple loci associated with disease susceptibility with genome-wide significance and other suggestive associations.^5, 7^ In particular, a strong HLA antigen association suggests that antigen-driven adaptive immune responses, possibly mediated by CD4^+^ T cells, contribute to disease pathogenesis.^8^ The intestinal microbiome has emerged as a key player in modulating mucosal immune responses, and dysbiosis has been linked to multiple immune disorders, including PSC.^9^ In a recent study, gnotobiotic mice inoculated with microbiota derived from patients with PSC exhibited T helper (Th) 17 cell responses in the liver and increased susceptibility to hepatobiliary injuries.^10^ These observations suggest that altered host-microbial interactions may result in immune dysregulation in PSC, leading to chronic inflammation and increased carcinogenesis.

Previous research observed distinct chemokine receptor profiles on circulating T cells in PSC-UC, confirming that specific changes in T-cell trafficking properties are involved in the pathogenesis of intestinal and liver inflammation. Studies have also found an accumulation of innate lymphoid cells (ILC) and increased Th1 responses in the colon of these patients, even in the presence of quiescent or mild activity.^11^ Furthermore, Th17 responses have been described in PSC,^12^ and increased levels of interleukin (IL)-17A and IL-22 in the colonic mucosa of patients with PSC-UC have been observed compared to levels in patients with UC alone (unpublished data). This information suggests that overactive type 1 and type 17 T-cell and unconventional lymphocyte responses may play a role in promoting intestinal inflammation in PSC-UC. However, nothing is known about the epithelial signature during chronic inflammation in PSC-UC and how this may impact neoplastic transformation.

The development of human organoids represents an invaluable tool to evaluate intestinal pathophysiology in vitro.^13^ In particular, human epithelial organoid cultures (EpOCs) can be derived ex vivo from intestinal crypts isolated from biopsies and surgical specimens and have been shown to retain the specificity of the intestinal segment of origin.^14^ Interestingly, a distinct transcriptional profile has been described in colonic EpOCs derived from patients with IBD, suggesting the presence of sustained changes in the epithelial stem cell compartment in these patients.^15^

This is the first study in which colonic organoids have been obtained from patients with PSC-UC. In particular, we cultured EpOCs derived from patients with PSC-UC, patients with UC alone, and healthy control patients to characterize the epithelial signature and evaluate the role of specific immune-epithelial interactions in the pathogenesis of increased risk of CRC in patients with PSC-UC.

## MATERIALS AND METHODS

### Patient Recruitment and Sample Collection

All patients and control patients were recruited from the Department of Gastroenterology, and samples were collected as part of the Translational Gastroenterology Unit biobank at the John Radcliffe Hospital in Oxford (UK) and part of the National Institute for Health Research Oxford Biomedical Research Centre IBD Cohort (REC 09/H1204/30) and the Oxford Biomedical Research Centre GI Illness Biobank (REC 16/YH/0247). Patients with a previous formal diagnosis of PSC-UC or UC according to clinical, radiological, endoscopic, and histological criteria were identified from the databases of our center and approached at outpatient clinic or endoscopy appointments to confirm consent and obtain colonic samples. Colonic biopsies were obtained from patients with PSC-UC and patients with UC alone undergoing colonoscopy for surveillance or disease activity and extension assessment and from healthy control patients undergoing colonoscopy for CRC screening or chronic abdominal pain/diarrhea with a macroscopically and microscopically normal colon. Endoscopic activity of PSC-UC and UC was defined by the Ulcerative Colitis Endoscopic Index of Severity score,^16^ and microscopic inflammation was defined by the Nancy score.^17^ Other clinical and demographic information, including current medications, were obtained from our clinical patient database.

### Crypt Isolation and Culture of EpOCs

The EpOCs were obtained as previously described.^13, 18^ Detailed protocols for human organoid culture were generously provided by Marc van de Wetering (Hubrecht Institute, Netherlands). In brief, colonic biopsies (n = 8-10) were washed in chelating buffer,^18^ incubated on ice for 20 minutes in 0.5M EDTA and 1mM dithiothreitol solution, vortexed and washed in Advanced Dulbecco’s Modified Eagle’s Medium with Nutrient Mixture Ham’s F-12 (Ad-DF; Gibco), and supplemented with 1% penicillin-streptomycin (Invitrogen), 1M Hepes (Invitrogen), and L-glutamine (Invitrogen; Ad-DF+++). Supernatants containing the epithelial crypts were collected and resuspended in reduced growth factor basement membrane extract (BME) type 2 (AMSBIO) to allow for the seeding of 100 to 300 crypts per 10 μL of BME in 48 well plates. Next, 200 μL of prewarmed organoid media^18^ was added to each well and kept at 37°C, 5% CO_2_ in a humidified incubator. Organoids were allowed to grow for 3 to 4 days before the first passage, then passaged once weekly for 2 weeks. The EpOCs were stimulated 2 days after second passage with IL-22 and interferon (IFN) γ (10 ng/mL; R&D Bio-Techne) for 24 hours (gene expression studies) and 15 minutes (confocal microscopy). The EpOCs were collected after 2 weeks of culture, embedded in paraffin to create a formalin fixed paraffin embedded (FFPE) block and cut at 5 μm for hematoxylin and eosin staining.

### RNA Extraction and Real-Time Quantitative Polymerase Chain Reaction Analysis

Total RNA was isolated from organoids using the RNeasy Minikit (Qiagen), including an on-column DNase digestion step (Qiagen). The cDNA was synthesized using the High Capacity cDNA Reverse Transcription Kit (Applied Biosystems) according to the manufacturer’s protocol. Gene expression was analyzed by real-time quantitative polymerase chain reaction (qPCR; TaqMan real-time PCR) and the Precision Fast qPCR Mastermix with ROX at a lower level with inert blue dye (Primer Design) and run on a ViiA7 Real-Time PCR System (Applied Biosystems). Data were analyzed using the ΔCT method [2^-ΔCT^ where ΔC_T_ = C_T(target gene)_ – C_T(endogenous control)_]. *RPLP0* was used as the endogenous reference gene for all qPCR analyses. The following TaqMan probes (Applied Biosystems) were used: *RPLP0* (Hs99999902_ m1), *FUT2* (Hs00382834_m1), *SOCS3* (Hs02330328_s1), *CIITA* (Hs00172106_m1), *CXCL2* (Hs00601975_m1), *OLFM4* (Hs00197437_m1), *LGR5* (Hs00969422_m1), *POU5F1* (Hs04260367_gh), *NANOG* (Hs04260366_g1), *STAT1* (Hs01014002_m1), *STAT3* (Hs01047580_m1), *MUC1* (Hs00159357_m1), *MUC2* (Hs00159374_m1), *IL22RA1* (Hs00222035_s1), *IRF1* (Hs00971965_m1), *IDO1* (Hs00984148_m1), *IFNGR1* (Hs00988304_m1), *IFNGR2* (Hs001942264_m1), *S100A9* (Hs00610058_m1), *CDH1* (Hs01023894_m1), *CDH2* (Hs00983056_m1), *SOCS1* (Hs00705164_s1), *PDL1* (Hs00204257_m1), and *PDL2* (Hs00228839_m1). For RNA expression analysis in colonic tissue, biopsies were collected in RNAprotect Tissue Tubes (Qiagen) and homogenized using Soft Tissue Homogenizing CK14 Kit vials (Stretton Scientific) in a Precellys 24 (Bertin Instruments) homogenizer (30 seconds at 3500 rpm). The RNA extraction, cDNA synthesis, and qPCR analysis were performed as described above.

### Immunofluorescence Imaging

Organoid samples were fixed with 4% paraformaldehyde, permeabilized using ice-cold methanol, blocked with 10% goat serum for 1 hour at room temperatire, and stained with the following primary antibodies overnight at 4°C: anti-E-cadherin clone 36/E-cadherin, (610181, BD Biosciences), anti-Phospho-Stat3 (Tyr705) clone D3A7, (9145, Cell Signalling), and anti-LGR5 (21833-1-AP, Proteintech). Goat anti-mouse IgG conjugated with Alexa Fluor 488 (A11001, Life Technologies) and goat anti-rabbit IgG conjugated with Alexa Fluor 555 (A32732, Life Technologies) were used as secondary antibodies. Nuclei were stained with Hoechst 33258 (Life Technologies). Images were acquired on an Olympus FV1200 IX83 Confocal System.

### RNA In Situ Hybridization

In situ hybridization for *OLFM4* was performed on colonic tissue sections using an RNAscope FFPE assay kit (Advanced Cell Diagnostics Inc., Hayward, CA). Following manufacturer protocol, 5 μm FFPE tissue sections were pretreated with H_2_O_2_ for blocking, antigen retrieval buffer, and protease digestion followed by hybridization with an *OLFM4* probe. Next, a horseradish peroxidase–based signal amplification system was hybridized before color development with 3,3’-di-aminobenzidine tetrahydrochloride. Samples were counterstained with hematoxylin and eosin and mounted with a mixture of distyrene (a polystyrene), a plasticiser (tricresyl phosphate), and xylene, called DPX. Positive staining was indicated by brown punctate dots in the nucleus and/or cytoplasm. Images were visualized and captured using the NanoZoomer S210 (Hamamatsu).

### Immunohistochemistry

Formalin-fixed paraffin-embedded tissue was sectioned at 5 microns and collected onto Superfrost glass slides. Tissue sections were dewaxed in xylene and rehydrated through alcohol to water. Endogenous peroxidase activity was blocked with 3% (v/v) H_2_O_2_ before masked antigens were retrieved by microwaving the tissue sections in target retrieval solution (Dako). Endogenous avidin and biotin were blocked (Vector Laboratories) before additional blocking of endogenous enzymes was performed using BloXall (Vector Laboratories). Tissue sections were further blocked with 10% (v/v) normal goat serum (Sigma-Aldrich) before being incubated overnight at 4°C in a humidified environment with anti-*OLFM4* XP rabbit monoclonal antibody (Clone D1E4M; Cell Signaling Technology) or normal rabbit immunoglobulin fraction (Dako).

After incubation, primary antibody labeling was detected with a biotinylated goat anti-rabbit IgG secondary antibody (Vector Laboratories). Tissue sections were then incubated with streptavidin horseradish peroxidase (Vector Laboratories) and signal-detected using diaminobenzidine (Vector Laboratories). Tissue sections were counterstained with Mayer’s Haematoxylin (Sigma-Aldrich) before being dehydrated through alcohol to xylene and mounted with DPX (Sigma-Aldrich) and coverslips applied. Images were collected on a Zeiss AxioScope A1 with ZEN Blue software (Carl Zeiss).

### Statistical Analysis

All statistical analyses were performed using GraphPad Prism (GraphPad Software, San Diego, CA) version 8. Differences between 2 groups were determined using the nonparametric Mann-Whitney U test: patients with PSC vs patients with UC, patients with PSC vs control patients, and patients with UC vs control patients. An ordinary 1-way analysis of variance (Tukey test as recommended) was used to show differences in cytokine stimulation between groups. For significance level, *P < 0.05 and **P < 0.01. Error bars represented the standard error of the mean.

## RESULTS

### Patient Characteristics

The characteristics of patients included in this study are shown in Table 1. The majority of patients with PSC-UC and those with UC only were male, whereas control individuals were equally distributed between male and female. Patients with PSC-UC were significantly younger than patients with UC (*P* < 0.04; Mann-Whitney *U* test), possibly reflecting the clinical indication for surveillance colonoscopy from diagnosis in patients with PSC-UC, whereas surveillance was indicated for long-standing colitis in patients with UC. No significant age difference was observed when comparing patients with colitis with control patients. As expected, the majority of patients with PSC-UC had extensive colitis, whereas disease distribution in patients with UC included both extensive and distal colitis. Colitis endoscopic activity was measured by the Ulcerative Colitis Endoscopic Index of Severity score and was comparable between patients with UC and patients with PSC-UC. The majority of intestinal samples showed quiescent or mild to moderate inflammation at microscopic examination, as measured by the Nancy score.

**TABLE 1.** Patient Characteristics

	PSC-UC	UC	Control Patients
**Colonic samples, n**	12	14	10
**Sex, male, n (%)**	8 (66)	10 (71)	5 (50)
**Age, y, median (range)**	31 (21-64)	58 (26-80)	52 (36-78)
**Small-duct PSC, n (%)**	1	—	—
**Liver cirrhosis**	0	—	—
**PSC-UC**	12	14	—
**UC duration, y, median (range)**	13.5 (5-33)	11 (1-47)	—
**UC extent**			
**Extensive, n (%)**	12	7	—
** Left-sided, n (%)**	0	6	—
** Proctitis, n (%)**	0	1	—
**UCEIS score**			—
** 0-2, n**	9	13	—
** 3-5, n**	3	1	—
** 6-8, n**	0	0	—
**Nancy score, average (range)**	1.3 (0-4)	0.8 (0-4)	—
**Treatment at time of sample collection**			
** 5-aminosalicylates, n (%)**	11	9	—
** Immunosuppressants, n (%)**	4	5	—
** Corticosteroids, n (%)**	3	0	—
** Anti-TNFs, (%)**	2	1	—
** Anti-integrin therapy, n (%)**	1	2	—
** Ursodeoxycholic acid, n (%)**	5	0	—

TNF indicates tumor necrosis factor drugs; UCEIS, Ulcerative Colitis Endoscopic Index of Severity.

In terms of liver disease, all except 1 patient with PSC-UC presented with large-duct PSC, and none had liver cirrhosis. At the time of sample collection, the majority of the patients with PSC-UC and UC were on aminosalicylates, and similar proportions were on immunosuppressants (ie, azathioprine) or biologic therapies. In addition, 42% of the patients with PSC-UC were also on ursodeoxycholic acid. Other concomitant treatments are noted in Table 1.

### EpOCs Showed Similar Expression of Genes Associated With Proliferation and Intestinal Cell Differentiation

**FIGURE 1.**
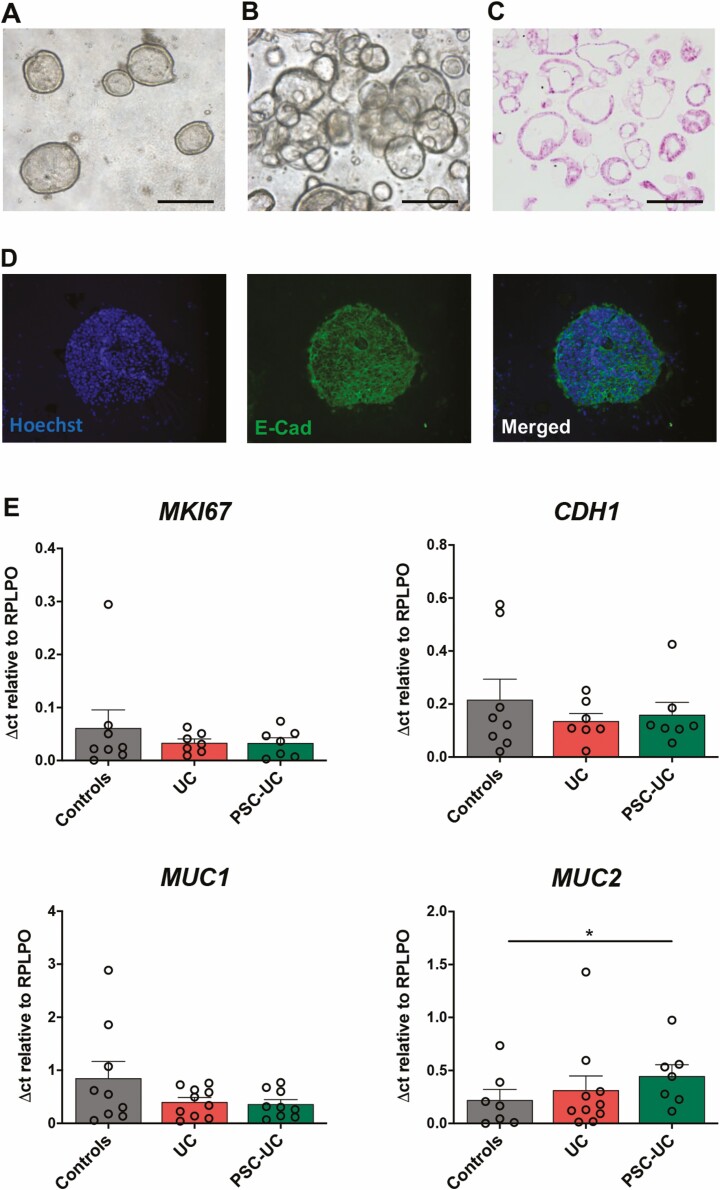
EpOCs show similar expression of genes associated with proliferation and intestinal cell differentiation. A-D, Morphological characterization of EpOCs derived from patient biopsies. A, Bright-field microscopy 48 hours after feeding. B, Two days after second passage, in BME and 24 well plates. C, Hematoxylin and eosin staining 2 days after second passage. and D, Immunofluorescent staining 2 days after second passage, with nuclei in blue and E-cadherin in green. Scale bar, 100 μm. E, mRNA expression of *MKI67, CDH1, MUC1,* and *MUC2* in EpOCs derived from control patients, patients with UC, and patients with PSC-UC. *P < 0.05. Error bars represent standard error of the mean.

We successfully grew EpOCs from colonic biopsies obtained at colonoscopy from control patients, patients with UC, and patients with PSC-UC (Fig. 1). Colonic crypts isolated from biopsies were seeded in BME-conditioned medium containing multiple growth factors, and the formation of 3-dimensional epithelial structures, or spheroids, was observed within 48 hours of culture (Fig. 1A). Once a week, spheroids were trypsinized to single-cell suspension, and cells were reseeded in BME. After 2 passages, we were able to obtain large numbers of EpOCs (Figs. 1B, C), which were characterized by a single layer of epithelial cells as confirmed by immunofluorescence (Fig. 1D).

To further characterize the EpOCs, we analyzed the expression of genes involved in proliferation and intestinal cell differentiation (Fig. 1E). We observed comparable expression of *MKI67,* a gene encoding for the nuclear protein Ki67, in EpOCs derived from all 3 patient groups, suggesting similar proliferative activity. The gene *CDH1,* encoding for the epithelial marker E-cadherin, was also equally expressed in EpOCs derived from the different groups. We observed no difference in the expression of the membrane-bound mucin *MUC1,* whereas *MUC2,* encoding for the main gel-forming mucin secreted by goblet cells in the mucus layers, was significantly overexpressed in PSC-UC derived EpOCs compared with those derived from control patients but not compared with those derived from patients with UC (Fig. 1E).

### Distinct Expression of Stemness-Associated Genes Observed in EpOCs Derived From Patients With PSC-UC

**FIGURE 2.**
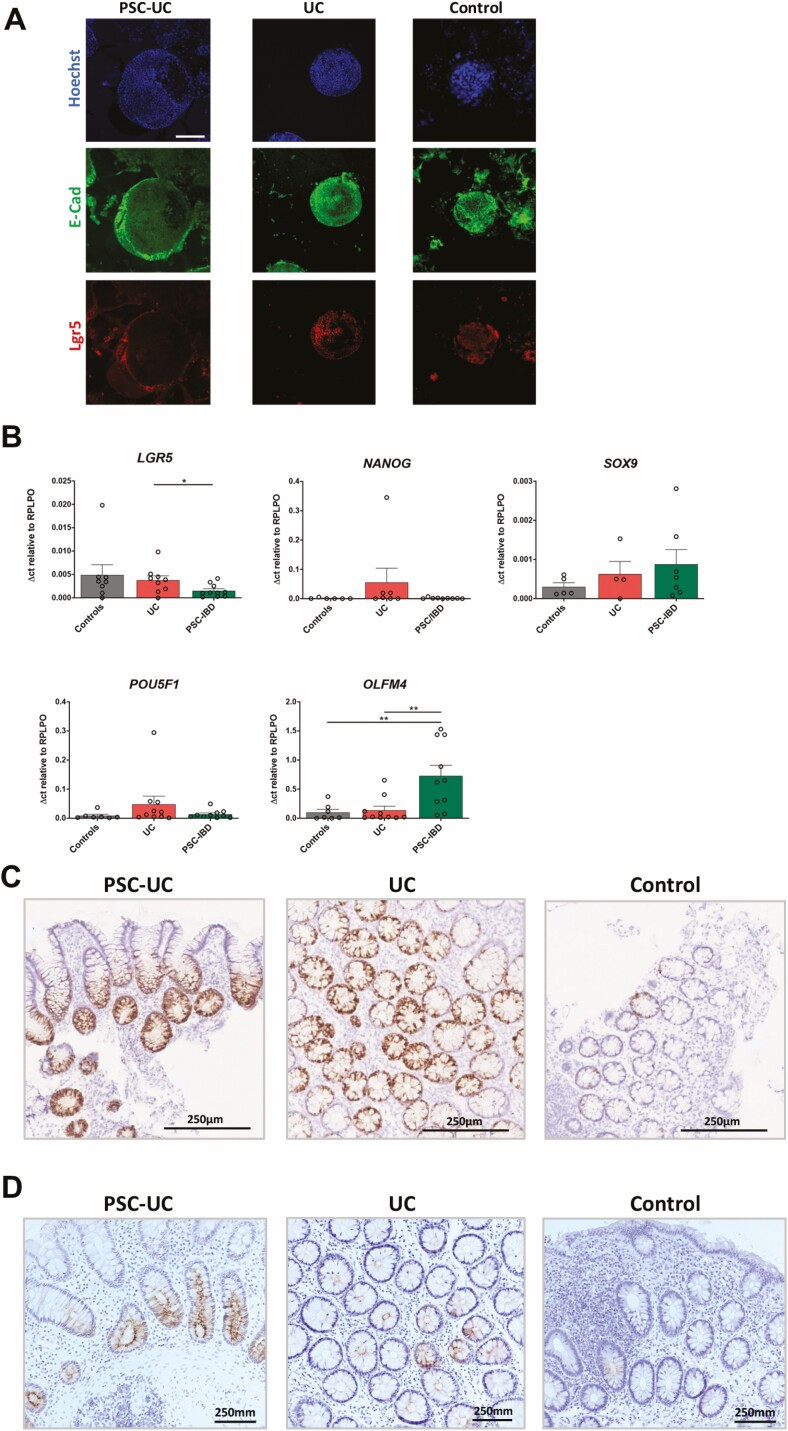
Distinct expression of stemness genes in EpOCs derived from patients with PSC-UC. A, Representative images of immunofluorescent staining of colonic organoids derived from patients with PSC-UC, patients with UC, and control patients with nuclei in blue, E-cadherin in green, and *LGR5* in red. Scale bar 100 μm. B, mRNA expression of *LGR5, NANOG, SOX9, POU5F1,* and *OLFM4* in EpOCs derived from 3 patient groups. *P < 0.05, **P < 0.01. Error bars represent standard error of the mean. C, Representative images of *OLFM4* transcript staining of noninflamed colon tissues derived from 3 patient groups. D, Representative images of *OLFM4* immunohistochemistry staining of noninflamed colon tissues derived from 3 patient groups. Scale bar 250 μm.

**FIGURE 3.**
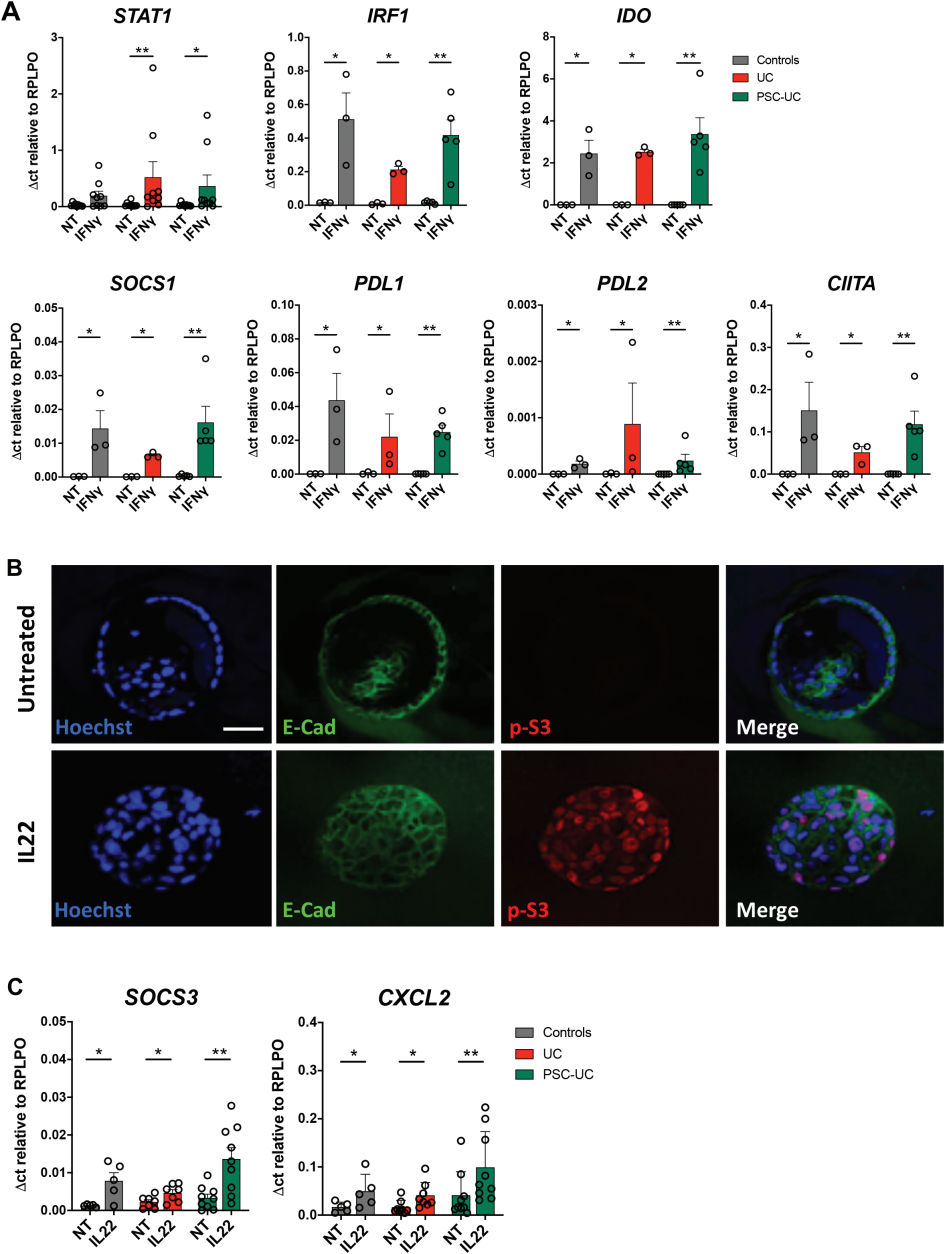
EpOCs respond to stimulation with cytokines. A, mRNA expression of *STAT1, IRF1, IDO, SOCS1, PDL1, PDL2,* and *CIITA* in EpOCs from control patients (grey bars), patients with UC (red bars), and patients with PSC-UC (green bars), with no treatment (NT) and after 24 hours of stimulation with IFNγ (10 ng/mL). B, Immunofluorescent staining, with nuclei in blue, E-cadherin in green, and phospho-*STAT3* (p-S3) in red in EpOCs untreated and stimulated with IL-22 for 15 minutes. Scale bar 100 μm. C, mRNA expression of *SOCS3* and *CXCL2* in EpOCs from control patients (grey bars), patients with UC (red bars), and patients with PSC-UC (green bars), with no treatment (NT) and after 24 hours of stimulation with IL-22 (10 ng/mL). Ordinary 1-way analysis of variance (Tukey test as recommended) was performed in the cytokine stimulation experiments. *P < 0.05, **P < 0.005. Error bars represent standard error of the mean.

**FIGURE 4.**
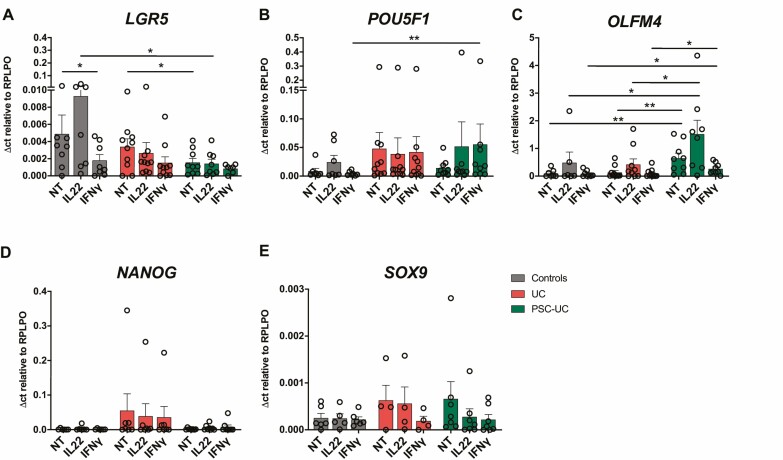
Cytokine stimulation regulating stem-cell gene expression in patient-derived EpOCs. mRNA expression of the stemness genes (A) *LGR5,* (B) *POU5F1,* (C) *OLFM4,* (D) *NANOG,* and (E) *SOX9* in EpOCs from control patients (grey bars), patients with UC (red bars), and patients with PSC-UC (green bars), with no treatment (NT) and after 24 hours of stimulation with IL-22 (10 ng/mL) and IFNγ (10 ng/ml). *P < 0.05, **P < 0.01. Error bars represent standard error of the mean.

**FIGURE 5.**
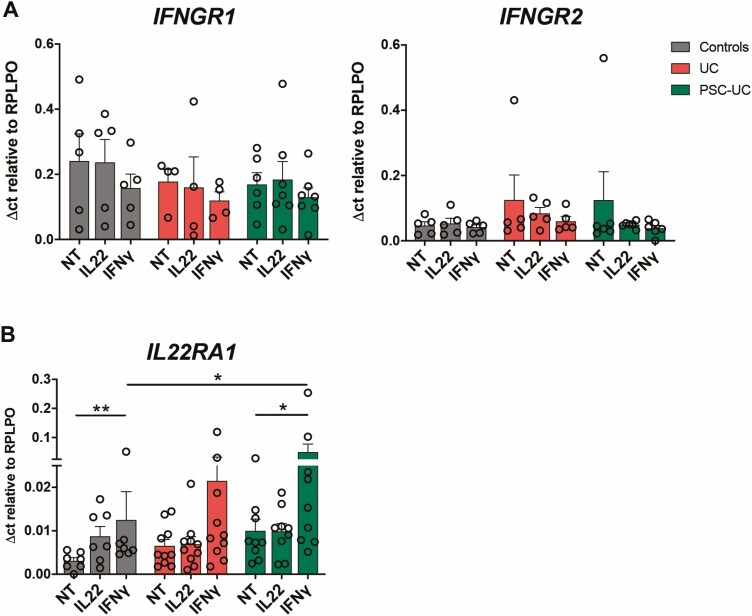
IFNγ stimulation inducing IL-22 responsiveness in EpOCs. mRNA expression of (A) *IFNGR1* and *IFNGR2* and (B) *IL22RA1* in EpOCs from control patients (grey bars), patients with UC (red bars), and patients with PSC-UC (green bars), with no treatment (NT) and after 24 hours of stimulation with IL-22 and IFNγ (10 ng/mL). *P < 0.05, **P < 0.00. Error bars represent standard error of the mean.

To assess epithelial stemness function, we evaluated markers of stem cell proliferation, self-renewal, and pluripotency in patient-derived EpOCs. The classical stem cell marker *LGR5* was expressed by EpOCs derived from patients with PSC-UC, UC, and control individuals, as observed by immunofluorescence staining (Fig. 2A). A significant decrease in the mRNA expression of *LGR5* was found in PSC-UC-derived EpOCs compared with those derived from patients with UC. No difference was observed in the expression of other stemness-associated genes, such as *NANOG, SOX9,* and *POU5F1,* between the 3 groups. Interestingly, a significantly increased expression of *OLFM4,* a member of the olfactomedin domain-containing protein family typically expressed in the stem cell niche, was found in EpOCs derived from patients with PSC-UC compared with both patients with UC and control patients (Fig. 2B). High levels of *OLFM4* expression were also observed by in situ hybridization (Fig. 2CSupplementary Figs. 1-3) and at the protein level by immunohistochemistry (Fig. 2DSupplementary Figs. 4-6) in colonic tissue from patients with PSC-UC, even in the presence of quiescent disease.

### Patient-Derived EpOCs Respond to Cytokine Stimulation Through *STAT* Phosphorylation and Gene Regulation

Previous research has shown an increased frequency of IFNγ-secreting T cells and an accumulation of ILC in the colon of patients with PSC-UC compared with patients with UC,^11^ and unpublished data have shown increased expression of Th1 and Th17 cytokines in the colon of these patients. To assess how inflammation can affect colonic epithelial function, we stimulated patient-derived EpOCs with IFNγ and IL-22. As expected, after IFNγ stimulation, we observed upregulation of IFNγ-induced genes, such as *STAT1, IRF1, IDO, SOCS1, PDL1, PDL2,* and *CIITA* (Fig. 3A) in the EpOCs derived from patients with UC/PSC-UC and control patients, with no difference observed between the groups. Stimulation with IL-22 resulted in *STAT3* phosphorylation (Fig. 3B) and the induction of IL-22-regulated genes, such as *SOCS3* and *CXCL2* (Fig. 3C). In addition, *SOCS3* expression after IL-22 stimulation was significantly higher in EpOCs from patients with PSC-UC than in EpOCS from patients with UC. However, no induction was observed for other IL-22-induced genes (Supplementary Fig. 7). These results suggest that EpOCs respond to IFNγ and IL-22 stimulation, providing a model to investigate immune-epithelial interactions.

### Differential Regulation of Stem Cell Gene Expression by Cytokine Stimulation in Patient-Derived EpOCs

When we looked at the expression of stemness-associated genes, we observed that IFNγ stimulation induced a significant downregulation of *LGR5* in EpOCs derived from control individuals and noted a similar trend in UC-derived EpOCs. The expression of *LGR5* in response to IL-22 stimulation was significantly higher in EpOCs derived from control patients when compared with PSC-UC derived EpOCS (Fig. 4A). The expression of *POU5F1* was significantly higher after IFNγ stimulation in PSC-UC-derived EpOCs compared with those of control patients (Fig. 4B). There was a trend toward a reduction of *OLFM4* expression after IFNγ stimulation in PSC-UC-derived organoids, but it remained higher than in EpOCs derived from patients with UC and from control individuals. Although a trend toward an induction of *OLFM4* was observed after IL-22 stimulation in all EpOCs, higher *OLFM4* expression was observed after IL-22 stimulation in EpOCs derived from patients with PSC-UC than in EpOCs derived from patients with UC and control patients (Fig. 4C). Cytokine stimulation with either IFNγ or IL-22 did not have any significant effect on the expression of *NANOG* and *SOX9* (Figs. 4D, E). These results indicate that immune responses involved in intestinal inflammation can affect the expression of genes associated to stemness and pluripotency in the intestinal epithelium. In particular, our data suggest that IFNγ induces the downregulation of *LGR5* and higher *POU5F1* expression in PSC-UC, whereas IL-22 results in higher levels of *OLFM4* in PSC-UC.

### IFNγ Stimulation Induces Increased IL-22 Responsiveness in Patient-Derived EpOCs

To assess epithelial responsiveness to the inflammatory milieu, we analyzed the expression of IFNγ and IL-22 cytokine receptor subunits on the EpOCs derived from the 3 groups. We found no difference in the expression of *IFNGR1* and *IFNGR2,* encoding for the 2 chains of the IFNγ receptor, between EpOCs derived from all 3 groups, and their expression was not affected by IFNγ or IL-22 stimulation (Fig. 5A). On the other hand, *IL22RA1* was significantly induced by IFNγ stimulation in the EpOCs from patients with PSC-UC and from control individuals, and a similar trend was also observed in UC-derived EpOCs (Fig. 5B). Moreover, the *IL22RA1* expression levels after IFNγ stimulation were significantly higher in the PSC-UC vs the control EpOCs, and a trend toward increased expression of *IL22RA1* was also found in colonic biopsies in PSC-UC (Supplementary Fig. 8). These results suggest that the high levels of IFNγ observed in PSC-UC^19^ may increase the epithelial responsiveness to IL-22, which in turn can induce *OLFM4* expression.

## DISCUSSION

Research has shown that PSC is a rare and severe hepatobiliary disease, leading to cirrhosis and liver failure, which is associated with IBD in 80% of patients, mostly diagnosed as UC. In addition, PSC-IBD is linked to a high risk of developing colorectal and hepatobiliary cancers, including cholangiocarcinoma, which together with liver failure are responsible for a high mortality rate in these patients. Unfortunately, the cause of PSC is unknown and there is no treatment available.

Colitis is often right-sided in PSC, and CRC more frequently arises from the right colon in these patients, suggesting that the underlying chronic inflammation may contribute to cancer initiation. Previous research noted an accumulation of Th1 and ILC in the colon of patients with PSC-UC,^11^ and other studies have implicated Th17 responses in the pathogenesis of PSC-IBD.^10, 19, 20^ In this study, we aimed to evaluate the role of immune-epithelial interactions in the pathogenesis of PSC-UC and the increased risk of CRC using patient-derived EpOCs.

We obtained EpOCs from colonic biopsies from control patients, patients with PSC-UC, and patients with UC with comparable disease activity, according to endoscopic and histological criteria. Organoids are 3-dimensional cell culture models or “mini organs” that are derived from stem cells.^14^ In intestinal organoids, *LGR5*^+^ stem cells can differentiate into enterocytes, goblet cells, Paneth cells, enteroendocrine cells, and enterochromaffin cells, rendering them representative of the original tissue and an invaluable tool for in vitro studies. Distinct transcriptomic profiles have been described in EpOCs derived from patients with UC compared with those derived from control patients, with a differential expression of genes involved in secretory and absorptive functions, antimicrobial defense, and inflammatory and carcinogenic pathways.^15, 21^ However, this is the first study to evaluate colonic EpOCs from patients with PSC-UC.

In this work, we observed a similar expression of genes associated with proliferation and differentiation in EpOCs derived from patients with UC, patients with PSC-UC, and control patients. However, a significantly higher expression of *MUC2* was found in EpOCs derived from patients with PSC-UC than in patients with UC and control patients. Studies have shown that *MUC2* is a mucin synthesized and secreted by goblet cells to form the inner mucous layer, a thick and dense barrier between the luminal microbiome and the epithelial cells, which contributes to mucosal defense.^22^ Mucin secretion is known to be regulated by bacterial products such as lipopolysaccharide and flagellin and by various cytokines.^23^ Dysbiosis has been previously described in PSC,^9^ and alterations of the mucous layer, through its effect on microbial-epithelial interactions, could contribute to the pathogenesis of intestinal inflammation in these patients. Interestingly, IL-22, a cytokine secreted mainly by Th17, Th22, and type 3 ILC, has been shown to play a central role in controlling mucin production.^24, 25^ IL-22^-/-^ mice show a significant reduction in *MUC1* and *MUC2* expression, and IL-22 stimulation can induce *MUC1* and *MUC2* expression in intestinal epithelial cells in mice.^26^ The increased Th17 and ILC responses in PSC may therefore contribute to mucosal barrier alterations in these patients.

We next evaluated the expression of genes associated with a stem-cell phenotype in EpOCs. Interestingly, we observed a significant downregulation of the classical stem-cell marker *LGR5* and an upregulation of *OLFM4,* which was highly expressed at the base of the crypts in patients with PSC-UC. *OLFM4* is a secreted glycoprotein identified as a signature gene for *LGR5*^+^ stem cells and is considered a robust marker of murine and human intestinal stem cells.^27^ Interestingly, *OLFM4* is also overexpressed in active IBD, and bacteria-led induction of *OLFM4* through Notch signaling has been previously reported in colon adenocarcinoma and mouse epithelial cell lines.^28, 29^ Furthermore, *OLFM4* is upregulated in colorectal adenomas^30^ and cancers of the gastrointestinal and hepatobiliary systems such as CRC, gastric cancer, and gall bladder cancer.^31^ Conversely, other studies have shown that *OLFM4* deletion in mice results in dextran sodium sulfate–induced intestinal inflammation and colon adenocarcinoma,^32^ and its downregulation has been observed in poorly differentiated and metastatic cancers.^31^ This disparity clearly indicates that sufficient levels of *OLFM4* are required to play a regulatory role in the intestine to maintain homeostasis and prevent dysbiosis.

To study immune-epithelial interactions, we stimulated EpOCs with cytokines previously associated with PSC-UC and with colitis-associated cancer, such as IFNγ and IL-22.^11, 33^ We confirmed that EpOCs respond to cytokine stimulation with *STAT* phosphorylation and gene induction. Interestingly, *LGR5* was downregulated in EpOCs derived from control patients after IFNγ stimulation, whereas higher *POU5F1* and *OLFM4* expression was observed in EpOCs derived from patients with PSC-UC after stimulation with IFNγ and IL-22, respectively.

Previously, IL-22 was shown to induce *OLFM4* in colon adenocarcinoma cell lines^29^; however, the opposite effects have also been reported, with IL-22 dampening the expression of *LGR5* and *OLFM4* in murine small bowel organoids.^34, 35^ Moreover, Th22 cells can promote CRC through the activation of *STAT3* and induction of the *DOT1L* complex, which regulates the expression of stem cell–associated genes, such as *NANOG, SOX2,* and *POU5F1.*^36^ However, in our study, *NANOG* and *POU5F1* were not significantly affected by IL-22 stimulation.

The cytokine IL-22 is considered as a double-edged sword in inflammation, with protective and pathogenic roles depending on the microenvironment. It is clearly involved in epithelial regeneration, and *IL22RA1* is upregulated in the transit-amplifying region of the intestinal crypts.^24, 36^ However, continuous IL-22 exposure in chronic inflammation may induce permanent changes in the stem niche that could contribute to epithelial transformation. Interestingly, we observed amplifying activity of Th1 and Th17 responses in the colonic epithelium, with IFNγ stimulation resulting in the upregulation of *IL22RA1.* A similar synergism between IFNγ and IL-22 receptors was previously found to play a central role in protection against viral infections in the intestine,^37^ highlighting the complex amplifying interaction between these evolution-related cytokines.

## CONCLUSIONS

Our study suggests that increased Th1 and Th17 responses in PSC may impact the intestinal epithelium, leading to alteration of the stem cell niche. The observed expansion of *OLFM4*^*+*^ and *POU5F1*^*+*^ transient amplifying cells may contribute to disease progression and increased cancer risk. Further studies are needed to characterize the stem cell niche in the intestinal and biliary epithelium in PSC and to assess whether targeting IL-12/IL-23 or more downstream cytokines, such as IFNγ or IL-22, may be beneficial for the treatment of these patients.

## Supplementary Material

izab025_suppl_Supplementary_Figure_1

izab025_suppl_Supplementary_Figure_2

izab025_suppl_Supplementary_Figure_3

izab025_suppl_Supplementary_Figure_4

izab025_suppl_Supplementary_Figure_5

izab025_suppl_Supplementary_Figure_6

izab025_suppl_Supplementary_Figure_7

izab025_suppl_Supplementary_Figure_8

## Notes

Author contributions: M.N. and S.S.B. designed, performed, and analyzed experiments and wrote the manuscript. Z.V. and S.B. performed experiments and provided technical support. S.T., C.V.A-C., and F.P. contributed to data interpretation and revised the manuscript. A.G. conceived and designed the project and contributed to data interpretation and revision of the manuscript.

Supported by: This work was supported by the Wellcome Trust (101734/Z/13/Z to A.G., 212240/Z/18/Z to F. P.), the National Institute for Health Research Research Capability Fund (to A.G.), the National Institute for Health Research Oxford Biomedical Research Centre (Gastroenterology and Mucosal Immunity Theme; to C.V.A-C., F.P., and S.T.), and Versus Arthritis (to S.B.).

## Abbreviations

BME: basement membrane extract
CRC: colorectal cancer
EpOCs: epithelial organoid cultures
IBD: inflammatory bowel disease
IFN: interferon
IL: interleukin
ILC: innate lymphoid cells
PSC: primary sclerosing cholangitis
PSC-UC: primary sclerosing cholangitis–associated ulcerative colitis
Th: T helper
UC: ulcerative colitis

## OXFORD IBD COHORT INVESTIGATORS

Phil Allan, Tim Ambrose, Adam Bailey, Ellie Barnes, Elizabeth Bird-Lieberman, Barbara Braden, Oliver Brain, Jane Collier, Emma Culver, James East, Lucy Howarth, Satish Keshav, Paul Klenerman, Simon Leedham, Rebecca Palmer, Astor Rodrigues, Jack Satsangi, Alison Simmons, Peter Sullivan, Holm Uhlig, and Alissa Walsh.
