# Supplementary figures and images for: Overexpression of Cancer-Associated Stem Cell Gene OLFM4 in the Colonic Epithelium of Patients With Primary Sclerosing Cholangitis
Source: Inflamm Bowel Dis. 2021 Feb 11;27(8):1316–27. doi: 10.1093/ibd/izab025 (PMC8314119; doi:10.1093/ibd/izab025)

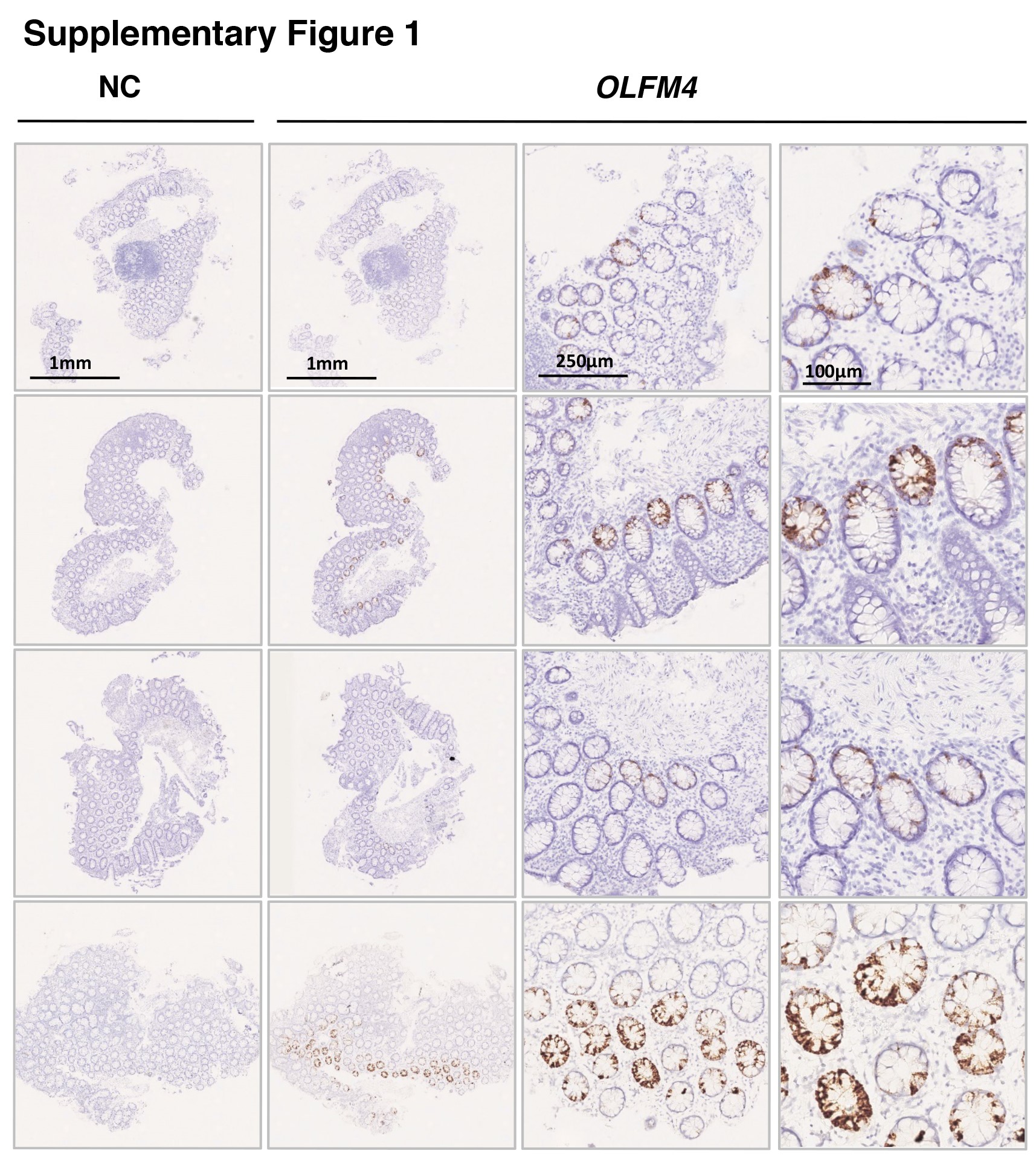

Supplement: izab025_suppl_Supplementary_Figure_1 [file izab025_suppl_supplementary_figure_1.jpeg]

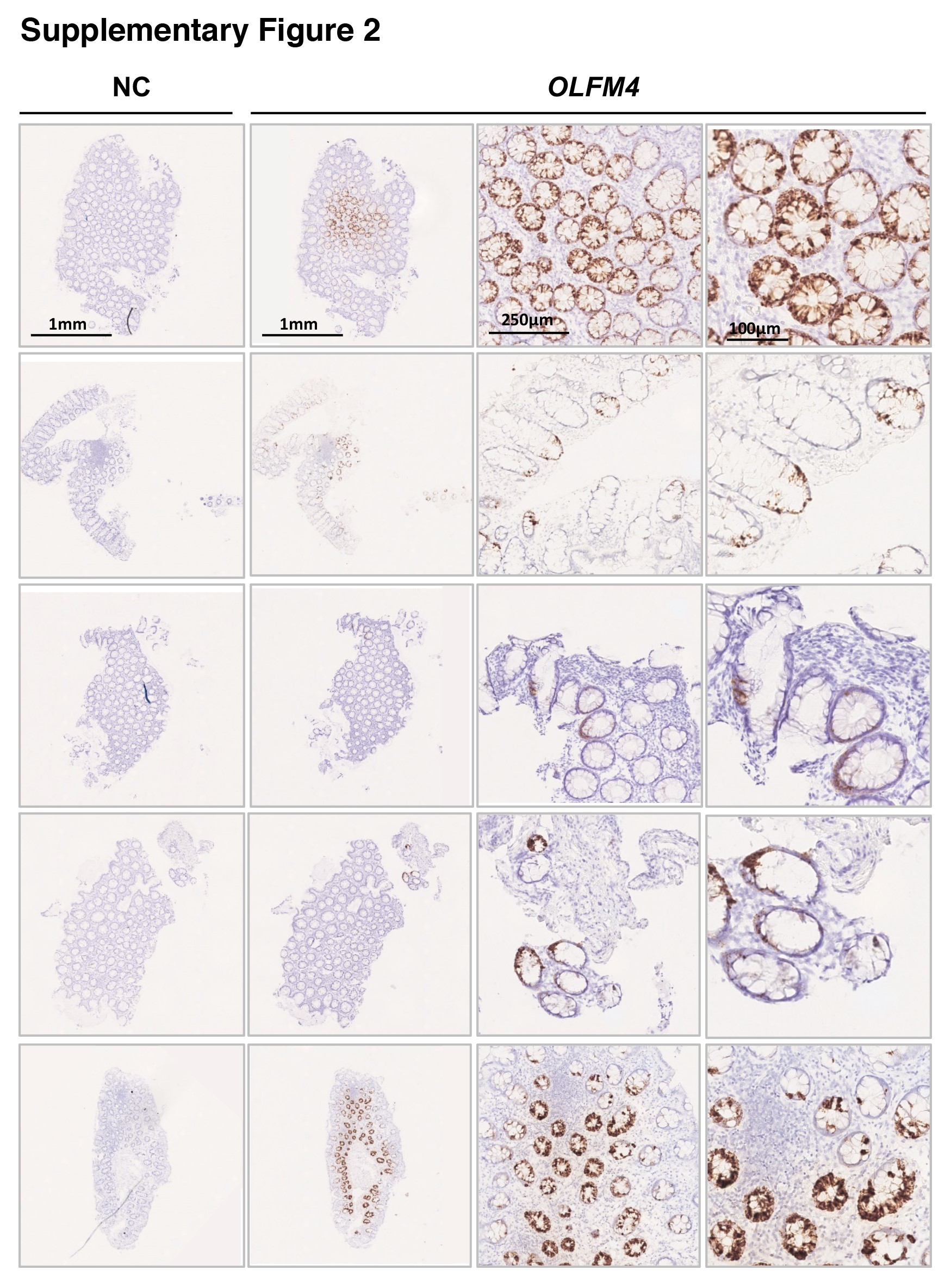

Supplement: izab025_suppl_Supplementary_Figure_2 [file izab025_suppl_supplementary_figure_2.jpeg]

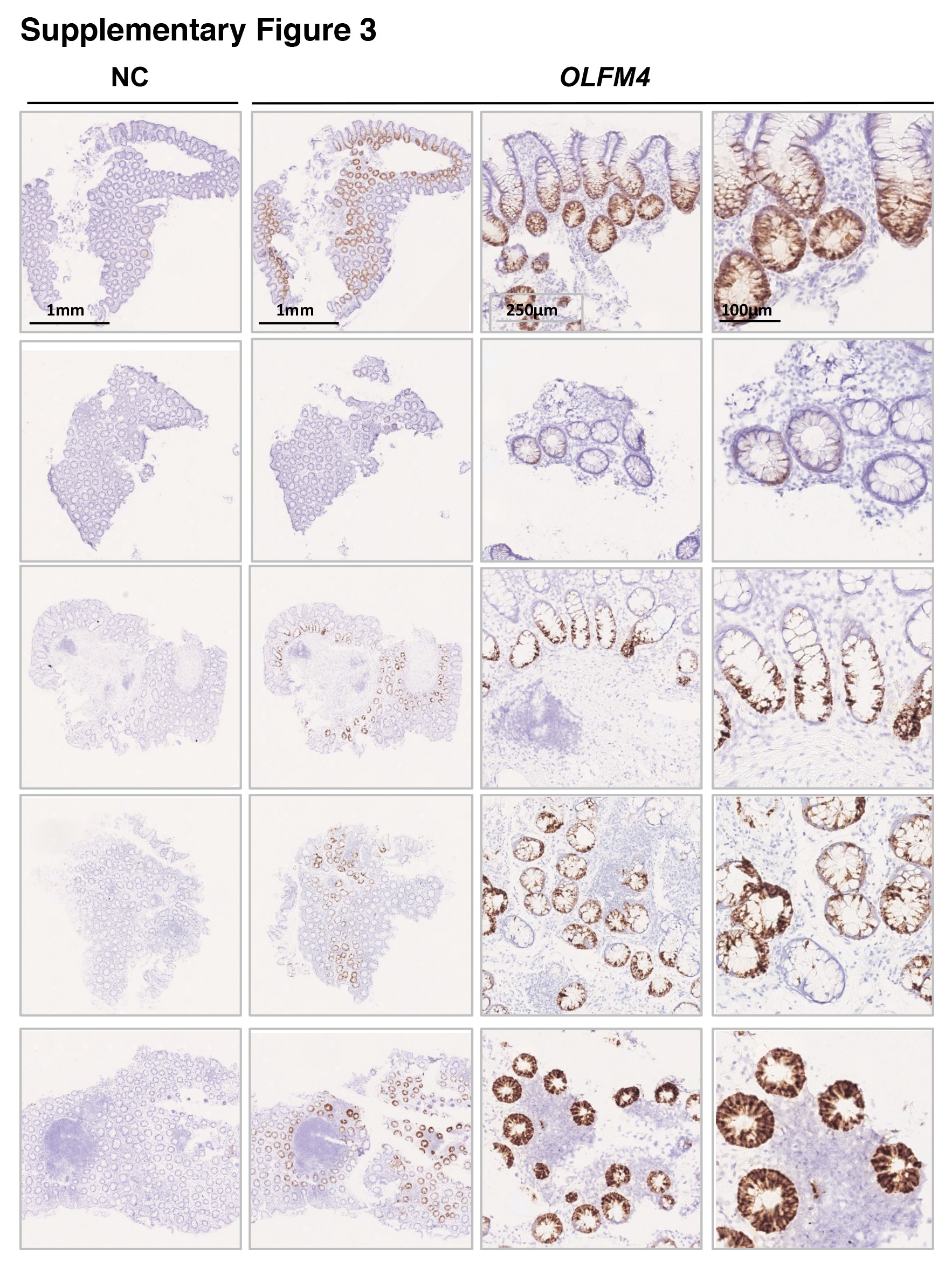

Supplement: izab025_suppl_Supplementary_Figure_3 [file izab025_suppl_supplementary_figure_3.jpeg]

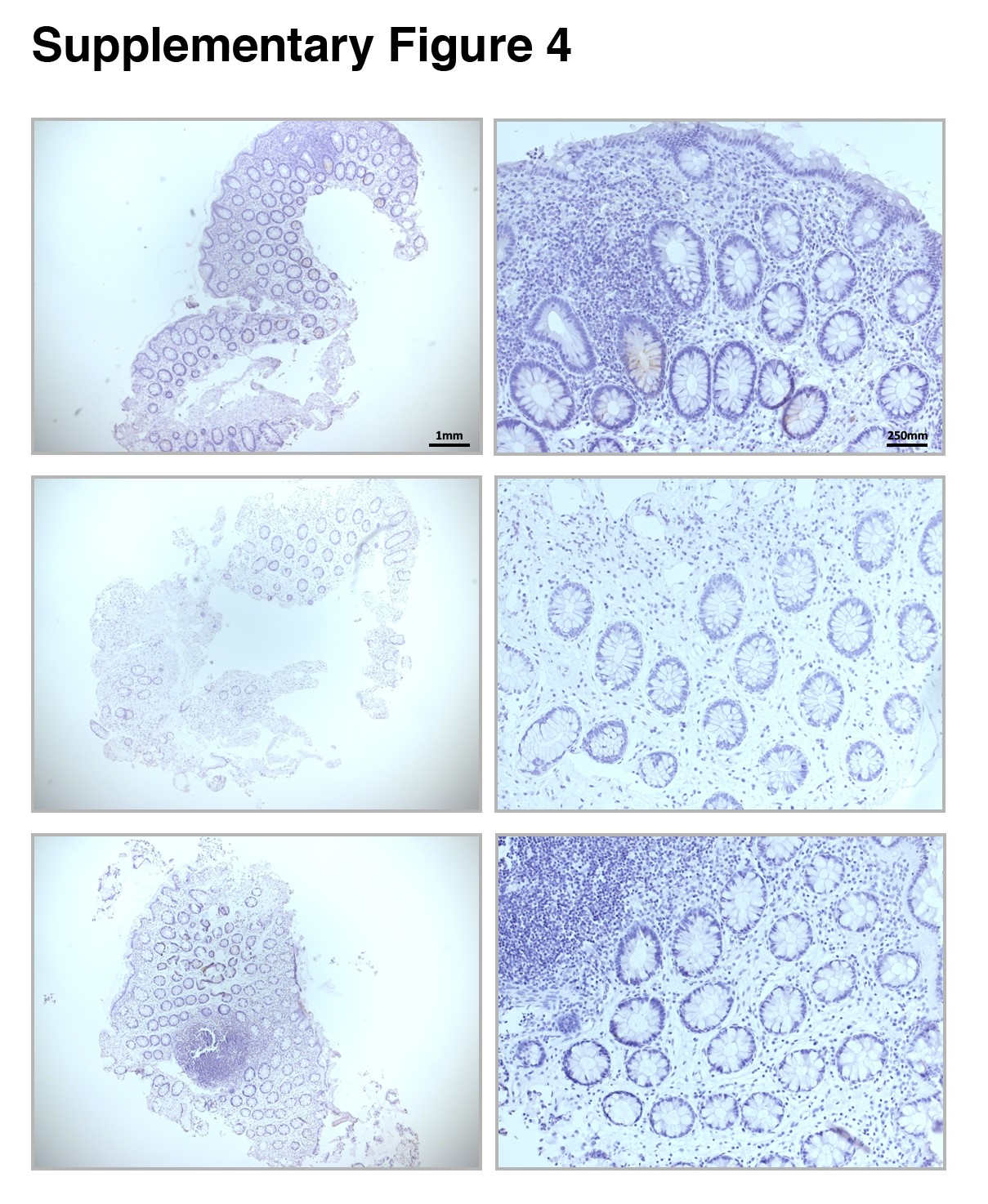

Supplement: izab025_suppl_Supplementary_Figure_4 [file izab025_suppl_supplementary_figure_4.jpeg]

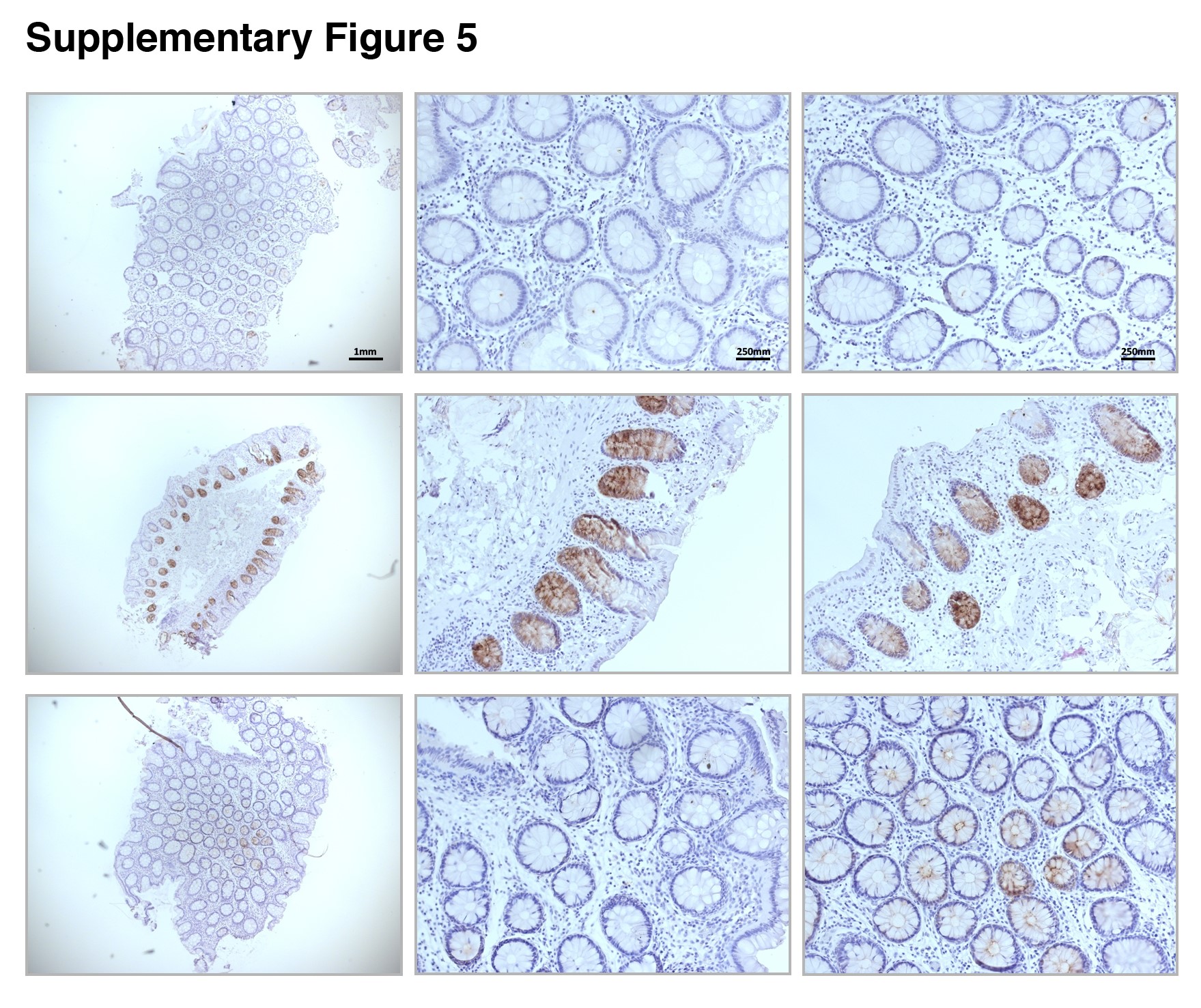

Supplement: izab025_suppl_Supplementary_Figure_5 [file izab025_suppl_supplementary_figure_5.jpeg]

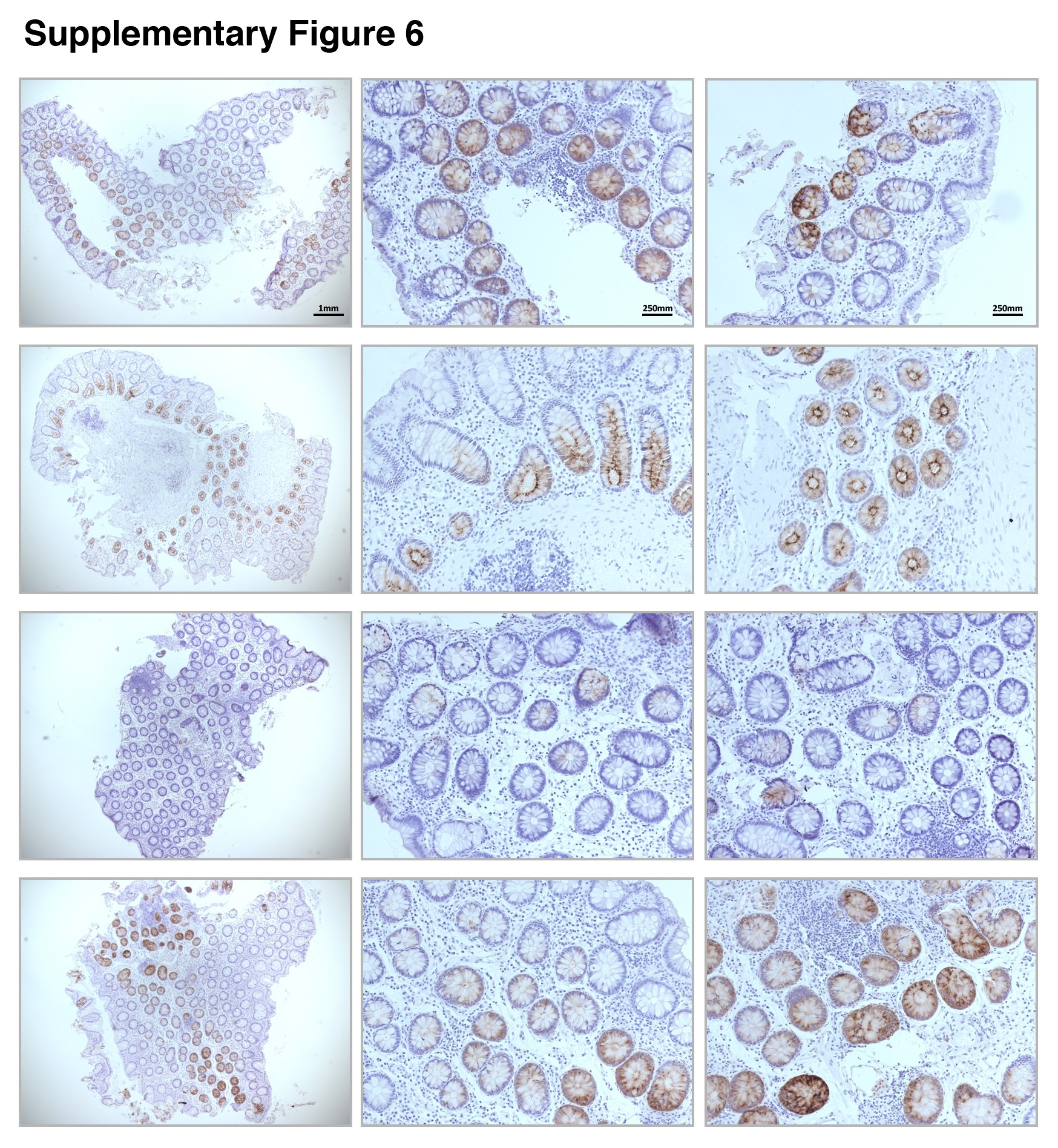

Supplement: izab025_suppl_Supplementary_Figure_6 [file izab025_suppl_supplementary_figure_6.jpeg]

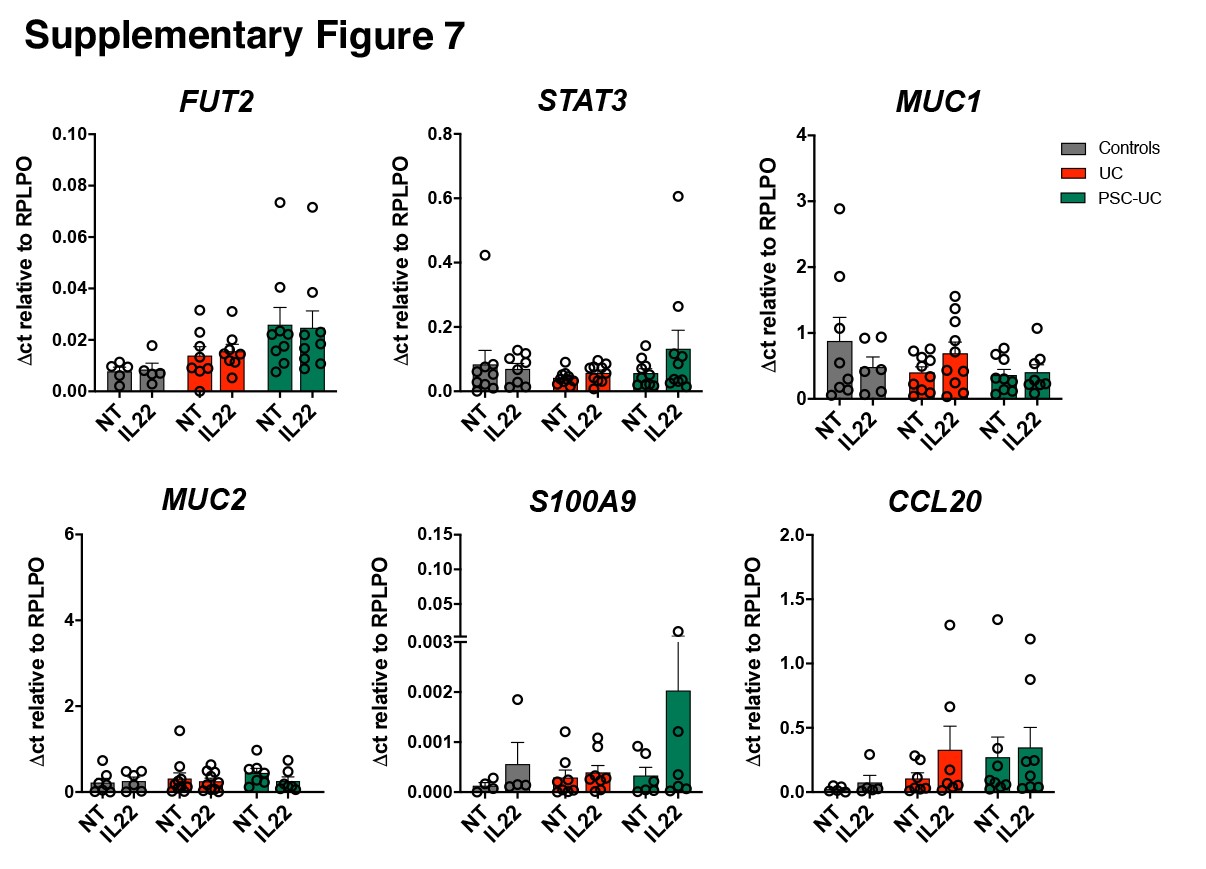

Supplement: izab025_suppl_Supplementary_Figure_7 [file izab025_suppl_supplementary_figure_7.jpeg]

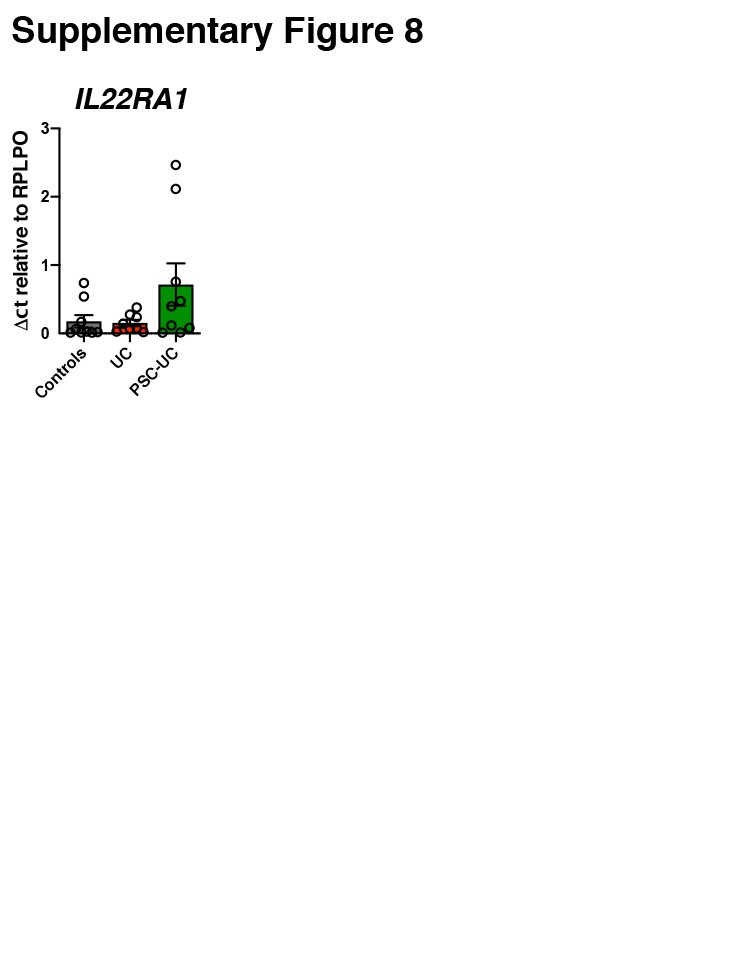

Supplement: izab025_suppl_Supplementary_Figure_8 [file izab025_suppl_supplementary_figure_8.jpeg]
